# Characterizing Deformability of Drug Resistant Patient-Derived Acute Lymphoblastic Leukemia (ALL) Cells Using Acoustic Tweezers

**DOI:** 10.1038/s41598-018-34024-3

**Published:** 2018-10-24

**Authors:** Hsiao-Chuan Liu, Eun Ji Gang, Hye Na Kim, Hae Gyun Lim, Hayong Jung, Ruimin Chen, Hisham Abdel-Azim, K. Kirk Shung, Yong-Mi Kim

**Affiliations:** 1 Department of Biomedical Engineering and NIH Ultrasonic Transducer Resource Center, University of Southern California, 1042 Downey Way, Los Angeles, CA 90089 USA; 2 Department of Pediatrics, Division of Hematology, Oncology, Blood and Marrow Transplantation, Children’s Hospital Los Angeles, University of Southern California, 4650 Sunset Blvd, Los Angeles, CA 90027 USA; 3 Department of Creative IT Engineering and Future IT Innovation Laboratory, Pohang University of Science and Technology, 77 Cheongam-ro, Nam-gu, Pohang, Gyeongbuk 37673 Republic of Korea

## Abstract

The role of cell mechanics in cancer cells is a novel research area that has resulted in the identification of new mechanisms of therapy resistance. Single beam acoustic (SBA) tweezers are a promising technology for the quantification of the mechanical phenotype of cells. Our previous study showed that SBA tweezers can be used to quantify the deformability of adherent breast cancer cell lines. The physical properties of patient-derived (primary) pre-B acute lymphoblastic leukemia (ALL) cells involved in chemotherapeutic resistance have not been widely investigated. Here, we demonstrate the feasibility of analyzing primary pre-B ALL cells from four cases using SBA tweezers. ALL cells showed increased deformability with increasing acoustic pressure of the SBA tweezers. Moreover, ALL cells that are resistant to chemotherapeutic drugs were more deformable than were untreated ALL cells. We demonstrated that SBA tweezers can quantify the deformability of nonadherent leukemia cells and discriminate this mechanical phenotype in chemotherapy-resistant leukemia cells in a contact- and label-free manner.

## Introduction

### Mechanical Property of Cancer Cells and Measurement Tools

Cellular biomechanics represent a significant characteristic in metastasis, as cancer cells are more deformable than normal cells, and this deformability correlates with increased metastatic potential^1^. Highly invasive cancer cells have been shown to be more compliant than weakly invasive cancer cells, allowing them to migrate easily^2–4^. As a result, the mechanical properties of a cell could potentially serve as useful biomarkers for the detection of metastatic cells in various cancers. Therefore, high-end biophysics technologies, including atomic force microscopy (AFM), optical tweezers, magnetic tweezers and acoustic tweezers, have been developed to measure the stiffness of a single cell^4^.

AFM is a powerful tool for the quantification of mechanical properties^2,5–9^. However, determining the elastic properties of suspended cells is challenging because of the lateral instability of cells under cantilevers^5^. Although AFM could quantify the deformability of suspended cells, a special mold was needed to immobilize the cells^5^. Other disadvantages of AFM include the risk that the sample could be regionally damaged by the pressure of the scanning cantilever, the high cost and the time-consuming process^10^.

Optical tweezers were developed by Ashkin in 1970^11^. Forces produced by the photons striking the cell along their propagation direction were found to be capable of exerting pressure on cells to produce a scattering force along the beam axis and a gradient force perpendicular to the beam axis^12^. For the past two decades, this technique has been used for single cell manipulation by a tightly focused laser beam due to the growing interest in cell mechanics^12^. However, optical tweezers might damage the cell structure and change its mechanical property by increasing the local temperature due to the increased laser power required to obtain strong optical forces. In addition, the trapping force of optical tweezers is only in the piconewton range, which may limit their applications^13,14^.

Magnetic tweezers have the unique advantages of a wide range of forces (10 pN–10 nN) and an infinite bandwidth^4^. The size of the nanoparticles determines the superparamagnetic or ferromagnetic properties of the particles. When a particle is placed in an external magnetic field, a magnetic moment is induced on the particle and causes it to move. A major drawback is that spherical magnetic beads must be implanted into the cytoplasm of a cell^15^.

Acoustic tweezers have been widely used since 1991^16^. Three types of major acoustic tweezers have been reported: bulk acoustic wave (BAW) tweezers by the B. Drinkwater group, surface acoustic wave (SAW) tweezers by the T. J. Huang group and single beam acoustic (SBA) tweezers by the K. Kirk Shung group^17^. BAW and SAW have been used in studies of cells/particles in manipulation, aggregation and gene expression analysis^18,19^, but they have not been used to evaluate the mechanical properties of cells since they require the use of one or more pairs of transducers^20^.

SBA tweezers represent a new technology for the manipulation of a single cell. The term “single beam” indicates that the tweezers are capable of manipulating a single cell or a particle with a single element transducer^21–23^. This technology was first theoretically and experimentally established by K. Kirk Shung’s group in 2005^21^. Compared with AFM, optical tweezers and magnetic tweezers, SBA tweezers have the following advantages in the measurement of mechanical properties: noncontact, low cost and high resolution^20^.

More recently, our previous study was a step forward in quantifying the mechanical properties of adherent cell lines using SBA tweezers, and we reported that highly invasive MDA-MB-231 cells are more deformable than are the weakly invasive MCF-7 and BT-474 cells^24,25^. However, quantifying the mechanical property in suspended cells is much harder than quantifying it in adherent cells because the adjacent cells around the targeted cell are easily disturbed by the trapping force. Interestingly, Byun *et al*. established a microchannel to assess the deformability of cancer cells, human lung adenocarcinoma cell line (H1975), and showed that cancer cells may display increased deformability and reduced friction, allowing invasive cancer cells to squeeze through tight spaces to enable metastasis. These findings suggest the possible role of increased deformability in the metastasis of cancer cells^1^.

### Deformability of Acute Lymphoblastic Leukemia (ALL) Cells

ALL is the most common type of childhood cancer and leukemia and the most widespread cause of cancer mortality, especially for those under 20 years of age. In the United States, approximately 6000 individuals are diagnosed with ALL annually, and half of them are children and teenagers^26,27^. Despite the 80% overall survival rate for children, drug resistance and subsequent relapse represent an important issue. Overall survival for adults with ALL is only 41%^28^. Therefore, drug resistance as well as the serious off-target toxicity of current therapies, which is occasionally dose-limiting, has prompted the quest for novel treatment strategies. Relapse of ALL or cancer in general is often attributed to poor prognosis-associated karyotypes and genes promoting drug resistance (REF). Recently, the mechanical properties of ALL cells have been explored as potential criteria to assess drug resistance or deformability in other hematopoietic cells, which could be potentially useful for improving diagnosis and relapse treatment^29^. M. E. Fay *et al*. reported that increased softness in normal leukocytes was related to leucocyte trafficking and increased blood counts^30^. M. J. Rosenbluth *et al*. used microfluidic biophysical flow cytometry to measure leukocytosis in the HL60 myeloid leukemia cell line and primary ALL cells taken at diagnosis^31^. A microfluidic technique was introduced recently by D. J. Hoelzle *et al*. to observe altered constriction and transit times of all-trans retinoid acid-treated HL60 cells^32^. In addition, this group reported that human breast cancer cells showed decreased deformability and increased invasiveness with the activation of beta-adrenergic signaling and that beta-adrenergic receptor activation reduces the deformability of ovarian, prostate, melanoma and leukemia cells^33^. K. D. Nyberg *et al*. described a quantitative method to determine the deformability of HL60 cells using flow cytometry^34^. A. Di Cerbo *et al*. established a micropipette aspiration technique to describe the effect of an antibiotic on K562 cells^35^. F. Lautenschlager *et al*. reported the softening of acute promyelocytic leukemia (APL) cells during differentiation that could not be altered by paclitaxel treatment through the stabilization of microtubules^36^. S. Khakshour *et al*. showed that the stiffness and viscosity of Jurkat T-ALL cells were increased after chemotherapy (artesunate) treatment, as demonstrated by oscillating optical tweezers^37,38^.

M. J. Michael *et al*. used AFM to measure the elastic modulus of leukemia cells plated in microfabricated wells needed to stabilize cells^5^. W. A. Lam *et al*. reported that the stiffness of acute myeloid leukemia (AML) and pre-B and T-ALL cell lines was increased by Daunorubicin/Dexamethasone treatment^6,39^ using AFM and that the freeze-thaw cycle does not alter cell stiffness^14^^.^ A. V. Muravyov *et al*. found decreased elastic properties of 15 patient-derived (primary) ALL cells after doxorubicin treatment for 1 or 24 hours using AFM^9^. Most of these findings are based on AFM studies or studies involving a flow cytometric approach. Therefore, an investigation of the mechanical property of ALL cells as a novel characteristic may improve our understanding of drug resistance.

We have used a coculture model of primary ALL cells with murine stromal cells (OP9) that allows us to model drug resistance *in vitro*^40^. When leukemia cell viability is maintained under continuous drug treatment before increasing again after withdrawing the drugs from the cell culture system, these cells are defined as drug resistant. Using this model of drug-resistant ALL cells, we investigated the mechanical properties of four primary pre-B ALL cells after chemotherapeutic treatment based on SBA tweezers and demonstrated that SBA tweezers can assess the deformability of ALL cells in suspension.

## Results

### Deformation of Primary Pre-B ALL Cells by the Acoustic Radiation Force of Single Beam Acoustic (SBA) Tweezers

**Figure 1:**
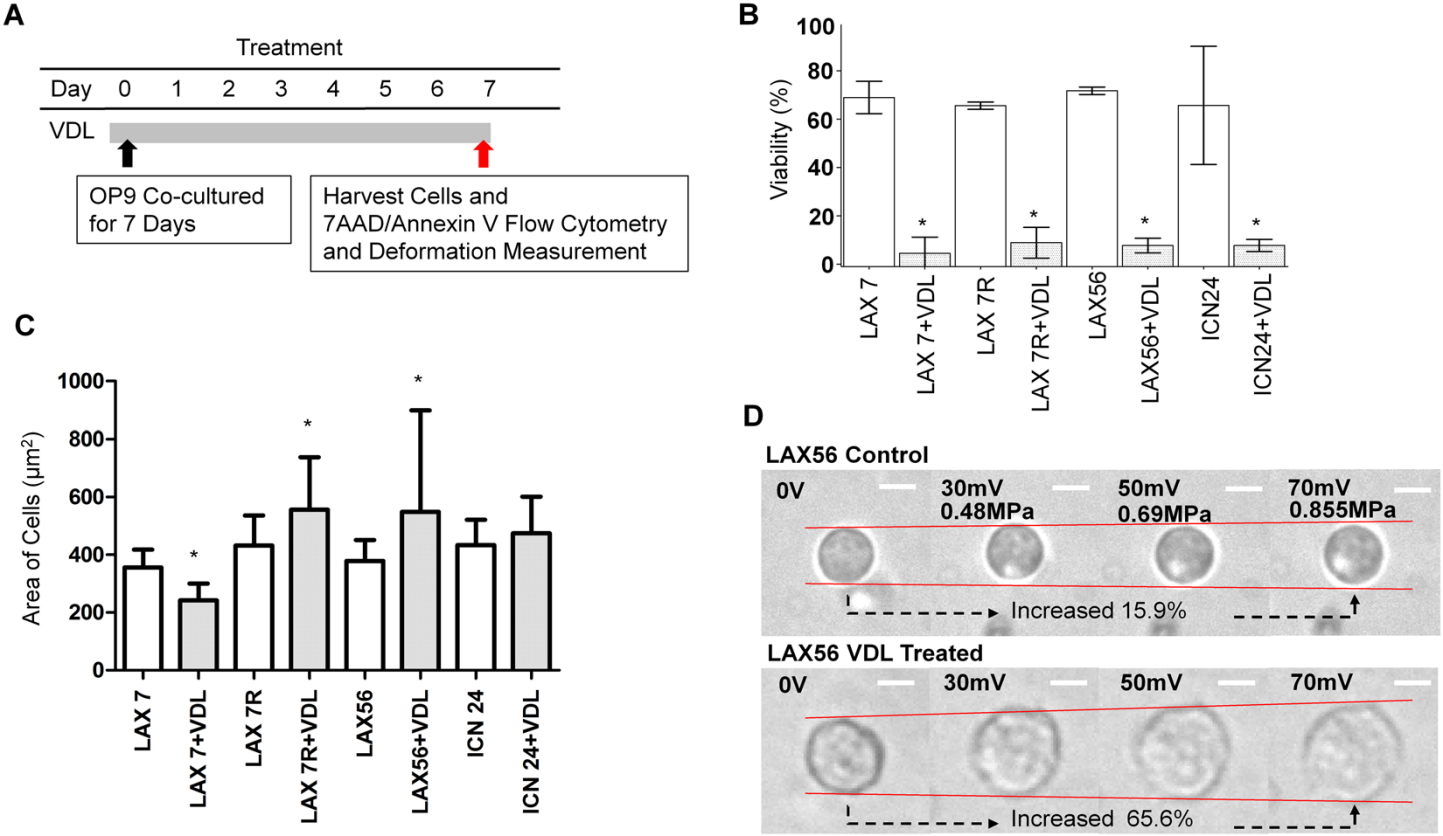
Area of cells and the viability of primary chemotherapy-resistant ALL cells. (**A**) Schematic of the experimental timeline. Primary ALL cells co-cultured with murine stromal OP-9 cells were treated with 0.005 µM vincristine, 0.05 nM dexamethasone and 0.0025 IU L-asparaginase (VDL 0.005) for 7 days or left untreated for 7 days. ALL cells were harvested on day 7 and analyzed by flow cytometry, microscopy for area of cell changes and acoustic trapping. (**B**) The viability of the control and VDL-treated cells on day 7 was measured by 7-AAD/Annexin V staining using flow cytometry before acoustic trapping. (**C**) Comparison of the area of cells treated for 7 days with VDL or left untreated for 7 days before acoustic trapping. (**D**) An example of the cell area measurement of ALL cells (LAX56) in the control group (upper row) and after 7 days of treatment with vincristine, dexamethasone and L-asparaginase (VDL) (lower row) before acoustic trapping. The white bars indicate 10 µm scale. *Indicates *p* < 0.05 compared with its control media group.

Four types of primary pre-B ALL cells (n = 133), LAX7, LAX7R, LAX56 and ICN24, were selected. Each type was divided into two groups: untreated (control) and drug-resistant ALL cells. For the control group, the numbers of LAX7, LAX7R, LAX56 and ICN24 cells were 11, 24, 19 and 21, respectively. In the drug-resistant group, the numbers of LAX7, LAX7R, LAX56 and ICN24 cells were 10, 10, 28 and 10, respectively. ALL cells were co-cultured with OP9 stromal cells. The drug-resistant ALL cells were derived from the cells after a 7-day treatment with the chemotherapeutic drug vincristine, dexamethasone and L-asparaginase (VDL). On day 7, the cells for both groups were harvested, and apoptosis was measured by 7-AAD/Annexin V flow cytometry, as illustrated in Fig. 1A. The viability of cells treated with VDL was significantly lower than the viability of the cells in the control group (Fig. 1B), indicating that most ALL cells were eradicated by the chemotherapeutic drugs. A small residual population that survived after the drug treatment was considered the drug-resistant population.

To investigate whether the deformability of a single primary ALL cell can be observed by the acoustic radiation force derived from acoustic trapping, we used SBA tweezers to assess the ALL cells. Each ALL cell was manipulated within the focal zone, and we scrutinized the changes in the transverse direction with varying voltages (see Methods section). Before the deformability experiment, the statistical results showed that the drug-resistant ALL cells were significantly larger than the ALL cells in the control group for three of the four types of ALL cells (*p* < 0.05 for LAX7, LAX7R and LAX56, *p* = 0.3014 for ICN24); in contrast, drug-resistant LAX7 cells appeared smaller than the control cells, as shown in Fig. 1C. Figure 1D shows an example of how the LAX56 ALL cells (top row, Supplementary video 1) and drug-resistant cells (bottom row, Supplementary video 2) were deformed by applying SBA tweezers and displays the deformability changes with varying voltages (varying acoustic pressures). The SBA tweezers could measure the mechanical property of suspended cells (ALL cells in the study). The procedure itself increased the area of the cells; under the given condition of an acoustic pressure of 0.855 MPa (70 mV from the signal generator) calculated by Equation (3), the deformability of the untreated LAX56 cells was increased to 15.9%. However, the drug-resistant LAX56 cells showed a much stronger increase in the deformation of cells, with an increase to 65.6%, as shown in Fig. 1D.

### The Mechanical Property of Primary Pre-B ALL Cells *versus* Drug-Resistant ALL Cells

**Figure 2:**
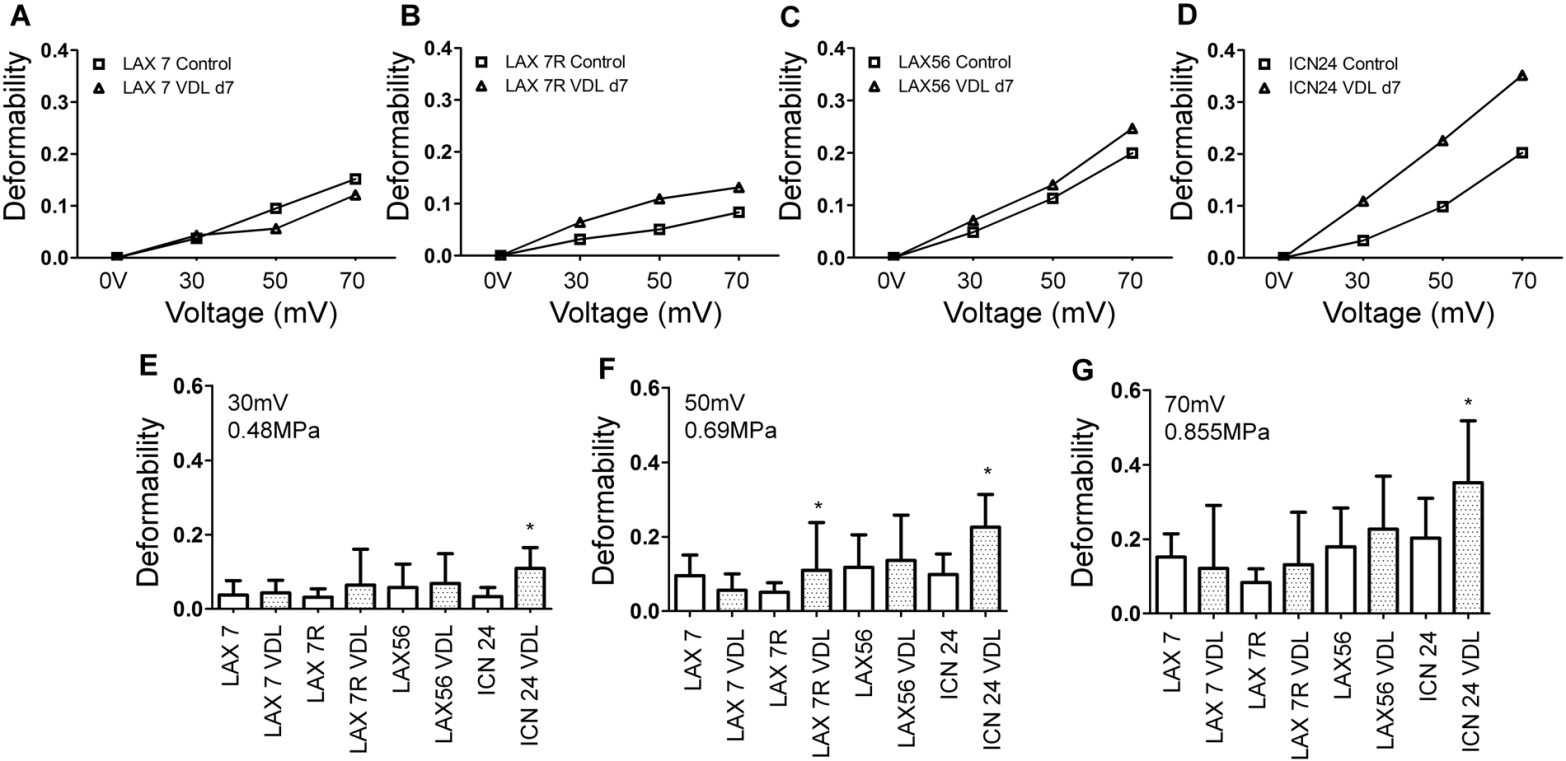
Increase in the deformation of chemotherapy-resistant primary pre-B ALL cells determined by acoustic trapping using SBA tweezers. Four cases of primary pre-B ALL, (**A**) LAX7, (**B**) LAX7R, (**C**) LAX56 and (**D**) ICN24, were treated with chemotherapy (vincristine, dexamethasone, L-asparaginase; VDL) or left untreated as controls. The deformation of single cells was caused using SBA tweezers with voltages of (**E**) 0.48 MPa (30 mV), (**F**) 0.69 MPa (50 mV) and (**G**) 0.855 MPa (70 mV). *Indicates p < 0.05 compared with the control media group.

The quantitative analysis of the mechanical property, deformability, of four types of primary pre-B ALL cells, LAX7, LAX7R, LAX56 and ICN24, was performed by using the SBA tweezer system. The deformability results revealed that this factor could discriminate between primary ALL and drug-resistant primary ALL cells (Fig. 2B–D). For LAX7R, at the acoustic pressure levels of 0.48 MPa (30 mV), 0.69 MPa (50 mV) and 0.855 MPa (70 mV), the mean deformability values of the cells were 0.031, 0.051 and 0.083 in the control group and 0.064, 0.11 and 0.13 in the VDL group, respectively. At the same pressure level, the mean values of the deformability of LAX56 were 0.048, 0.113 and 0.2 in the control group and 0.071, 0.139 and 0.246 in the VDL group, respectively. Regarding the mean deformability of ICN24, the values were elevated from 0.033 and 0.098 to 0.202 in the control group and from 0.109 and 0.226 to 0.352 in the VDL group. The opposite results were observed for the mechanical property in LAX7 ALL cells; the deformation of drug-resistant LAX7 cells was smaller than that of the control cells as the acoustic pressure was higher than 0.48 MPa (30 mV), which is illustrated in Fig. 2A. Consistent results were also observed using microscopy, as shown in Fig. 1C; the area of the drug-resistant LAX7 ALL cells was smaller than that of the control cells before the deformability experiment.

The deformability of four types of untreated and drug-resistant ALL cells, LAX7, LAX7R, LAX56 and ICN24, was obtained at several acoustic pressures: 0.48 MPa (30 mV), 0.69 MPa (50 mV) and 0.855 MPa (70 mV) (Fig. 2E–G). For the acoustic pressure at 0.48 MPa, the mean deformability of ICN24 ALL cell pairs revealed a significant difference (*p* < 0.0001), whereas the other three types of drug-resistant ALL cells showed less stiffness than the control cells did. For the acoustic pressure at 0.69 MPa (50 mV), the LAX7R pair and ICN24 pair displayed significant differences, with *p* values of 0.036 and < 0.0001, respectively. Although there was no significant difference for the LAX56 pair, the stiffness of drug-resistant LAX56 ALL cells was examined and shown to have a mean deformability of 0.136, while the LAX56 control cells had a mean deformability of 0.118. However, drug-resistant LAX7 ALL cells still exhibited greater stiffness than did LAX7 ALL control cells, as indicated by the mean deformability shown in Fig. 2F. For the acoustic pressure at 0.855 MPa (70 mV), a larger deformability was observed for three of the four types of drug-resistant ALL cells, except LAX7 ALL cells, than that for the control cells, and the ICN24 pair was significantly different (*p* = 0.0052). The results showed that ICN24 cells had a highly sensitive mechanical property, with significant differences (*p* < 0.05) observed when the acoustic radiation force was exerted at all three different acoustic pressures. In summary, we demonstrated that the drug-resistant primary pre-B ALL cells exhibited a greater deformability than the untreated ALL cells did. SBA tweezers can discriminate the mechanical property of drug-resistant ALL cells from that of untreated ALL cells and are capable of exploring the mechanical property of suspended cells.

## Discussion

Monitoring ALL cells allows a precise assessment of the early treatment response and relapse detection. Common methods to detect leukemia cells in the peripheral blood or bone marrow are flow cytometric detection of abnormal immunophenotypes, polymerase chain reaction (PCR) amplification of antigen-receptor genes and PCR amplification of fusion transcripts^41^. In a previous study, SBA tweezers were used as a novel method to quantify a mechanical property, deformability, of adherent cells^24^. However, using AFM to quantify the mechanical property of suspended cells is difficult because of the lateral instability of cells under cantilevers^5^. One solution involved microwells that were fabricated to stabilize the cells pushed into wells by the cantilever tip. In this report, we demonstrated that SBA tweezers could quantify the mechanical properties of suspended cells. The application of SBA tweezers have become more prominent in research on cell mechanics.

Primary pre-B ALL cells from four cases were continuously treated *in vitro* with VDL for 7 days to eradicate the cells, and the few residual viable leukemia cells were considered drug-resistant cells. In this study, these drug-resistant ALL cells revealed a greater compliance than did the cells in the control media (Fig. 2). Previous studies of cell stiffness indicated that ovarian cancer cells are generally softer than nonmalignant ovarian epithelial cells based on the AFM method, and some cell types with high metastatic potential increased deformability possibly to efficiently squeeze through tight spaces^2^. Additionally, A. V. Muravyov *et al*. reported that cell stiffness is inversely proportional to drug concentration^9^. The researchers noted that the low membrane rigidity of surviving ALL cells is due to the high concentrations of doxorubicin in the medium. These results are in accordance with our findings. The drug-resistant ALL cells in VDL after the 7-day treatment showed more deformation than did the untreated ALL cells. Although performing a chemotherapy dose-response study for the deformability assay would have been beneficial, we selected one dose of chemotherapeutic drugs, as it significantly decreases the viability of ALL cells compared to that of control-treated cells and then results in a plateau, thus defining the drug-resistant state. In addition, this dose allowed us to determine the number of drug-resistant cells needed for the cell deformability assay.

Interestingly, LAX7 ALL cells showed opposite effects: the deformability of drug-resistant cells was smaller than that of control cells. LAX7 is a matched diagnosis sample of the relapsed sample LAX7R. LAX7 was more sensitive to chemotherapeutic drugs than were the subsequent LAX7R samples; therefore, the characterization of deformability may reflect the chemosensitivity of LAX7 cells. Some studies have indicated that the deformability of cells was not always correlated with drug resistance or malignancy. Patient chronic lymphocytic leukemia (CLL) cells had a lower deformability than did those from healthy samples based on a microfluidic device^42^. HL60 AML cells and Jurkat T-ALL cells also showed low deformability after 15 min of dexamethasone or daunorubicin drug treatment^6^. However, using a cell line propagated for years *in vitro* may not reflect the physical status of patient cells, and the increase in stiffness could depend on the chemotherapy type^6^. In this study, a 7-day treatment of four primary pre-B ALL cells with VDL was required to reflect the actual response of the cells. However, studies focusing on the mechanical properties of chemosensitive cells are needed to explore in the further works.

A relatively high standard deviation could not be avoided and could decrease the significance of the results due to the very small scale of the cells and highly sensitive acoustic tweezer system. Similar results were reported in previous studies of cell deformation^5,24^. AFM is a well-established tool for the study of intrinsic cellular mechanical properties due to its high sensitivity, with a resolution of 0.1–0.5 nm in the vertical direction and 1–5 nm in the horizontal direction in air^43,44^. However, the lateral instability of suspended cells under cantilevers will be an issue in the measurement of mechanical properties; thus, extra tools may be needed to immobilize suspended cells and to increase throughput^5,6^. Additionally, the local stress-strain relationship under compression cannot be represented by a simple linear relationship using AFM due to the complex, heterogeneous media of cells^14^. Therefore, simplistic mechanical models are generally used to characterize cell mechanics by measuring the global elasticity of the cells^14,24,45^. A previous study reported that the different deformability of normal hematopoietic cells and leukemic cells could be characterized by stretching cells using optical tweezers^45^, whereas relatively high laser powers of approximately 800 mW could only produce a force with hundreds of piconewtons^24^. In this study, we demonstrated that SBA tweezers could quantify the global elasticity property of suspended cells by compression by axial acoustic pressure without extra tools. Although conventional approaches, such as AFM, micropipette aspiration, microbead rheometry, to measure the mechanical properties of a signal cell are widely used, low or potentially high throughput could still need to be considered in the experiment^29^. Even though measuring mechanical properties with high throughput can be achieved by using microfluidic channels^29,32^, compatibility, microfluidic channels designed to very specific objects, ill-suited to mass production and complex operational control are the challenges^46,47^. In addition, the interfacial tension and surface forces between immiscible fluids dominate gravitational forces once cross-sectional dimensions are down to hundreds of micrometers, which will lead to an interfacial (Rayleigh-Plateau) instability^46^. In contrast, SBA tweezers may have a relatively high throughput if delicate transducers with a very high frequency and a small *f*-number are used, and are capable of exerting on several sizes of cells. SBA tweezers could represent a promising method in the quantification of suspended cells in cell mechanics.

## Conclusion

In summary, we demonstrated that the acoustic trapping technology based on SBA tweezers is a promising tool in the quantification of the mechanical property of suspended cells. The deformability of four types of primary pre-B ALL cells, LAX7, LAX7R, LAX56 and ICN24 (n = 133 ALL cells), was assessed by SBA tweezers. Each ALL cells was divided into two groups: untreated ALL cells and drug-resistant ALL cells after chemotherapeutic treatment. The results revealed that SBA tweezers can quantify the mechanical properties of suspended cells. Second, the drug-resistant ALL cells were more deformable than were the untreated ALL cells. Moreover, SBA tweezers could determine the mechanical property of drug-resistant ALL cells and distinguish them from untreated ALL cells. Analysis of a certain amount of primary cells (n = 133) in the experiment could represent a step forward in clinical applications. In future studies, more mechanical parameters, such as Young’s modulus and Poisson’s ratio, could be assessed by SBA tweezers. We are developing a new method to measure Young’s modulus of ALL cells with SBA tweezers as the basis. This method will involve new ideas that we conceived recently. Briefly the new idea is that we will perform SBA tweezer measurements on agarose beads which sizes are similar to the real cells. The Young’s modules of agarose beads will be obtained by using AFM as the gold standard. Based on those data, the Young’s modulus of leukemia cells can be inferred from the results from the agarose beads. In addition, ALL cells at different phases of treatment and cells exposed to different drug concentrations will be another intriguing points to explore the drug resistance of cells with acoustic tweezer technology. More recently, the local mechanical property and cellular behavior were found to be affected by the cytoskeleton, representing both internal and external physical forces^48^. The mechanism of cell deformability and its links to drug resistance will need to be further explored.

## Methods

### Primary Pre-B ALL Cell Culture and Treatment with Chemotherapeutic Agents

Primary pre-B ALL cells, LAX7, LAX7R, LAX56 and ICN24, were cocultured as described previously^40,49^ with irradiated murine stromal OP-9 cells on tissue culture plates in minimum essential medium-α (MEM-α) supplemented with 20% heat-inactivated fetal bovine serum (FBS, Invitrogen), 2 mM L-glutamine (Invitrogen), 100 U/mL penicillin and 100 ${\rm{\mu }}$ g/mL streptomycin (Invitrogen) (α20, complete culture media). All the cells were incubated at 37 °C under a 5% CO_2_ atmosphere.

ALL cells were treated with VDL (V: 0.005 µM vincristine; D: 0.05 nM dexamethasone and L: 0.0025 IU L-asparaginase) in the presence of the OP-9 stromal layer for 7 days. Control cells were left untreated but were also cultured for 7 days with OP9 cells. ALL cells were then harvested by centrifugation at 500 rcf (relative centrifugal force) for 5 min and re-suspended in DPBS (Dulbecco’s phosphate-buffered saline, Invitrogen) containing 2% FBS at a concentration of 0.05 × 10^6^ cells/mL prior to deformability testing. More details are given in previous papers^40,49^.

### Apoptosis Assessment by Flow Cytometry

Apoptosis of ALL cells was determined by 7-AAD/Annexin V-PE staining (Biolegend) using flow cytometry (FACSCalibur, Becton Dickinson). Flow cytometry data were analyzed by FlowJo^®^ software (FlowJo LLC). The apoptotic fraction of cells was defined as the Annexin V^+^ cells, including single Annexin V^+^ (early apoptosis) and Annexin V^+^/7-AAD^+^ (late apoptosis) cells.

### The Principle of SBA Tweezers

**Figure 3:**
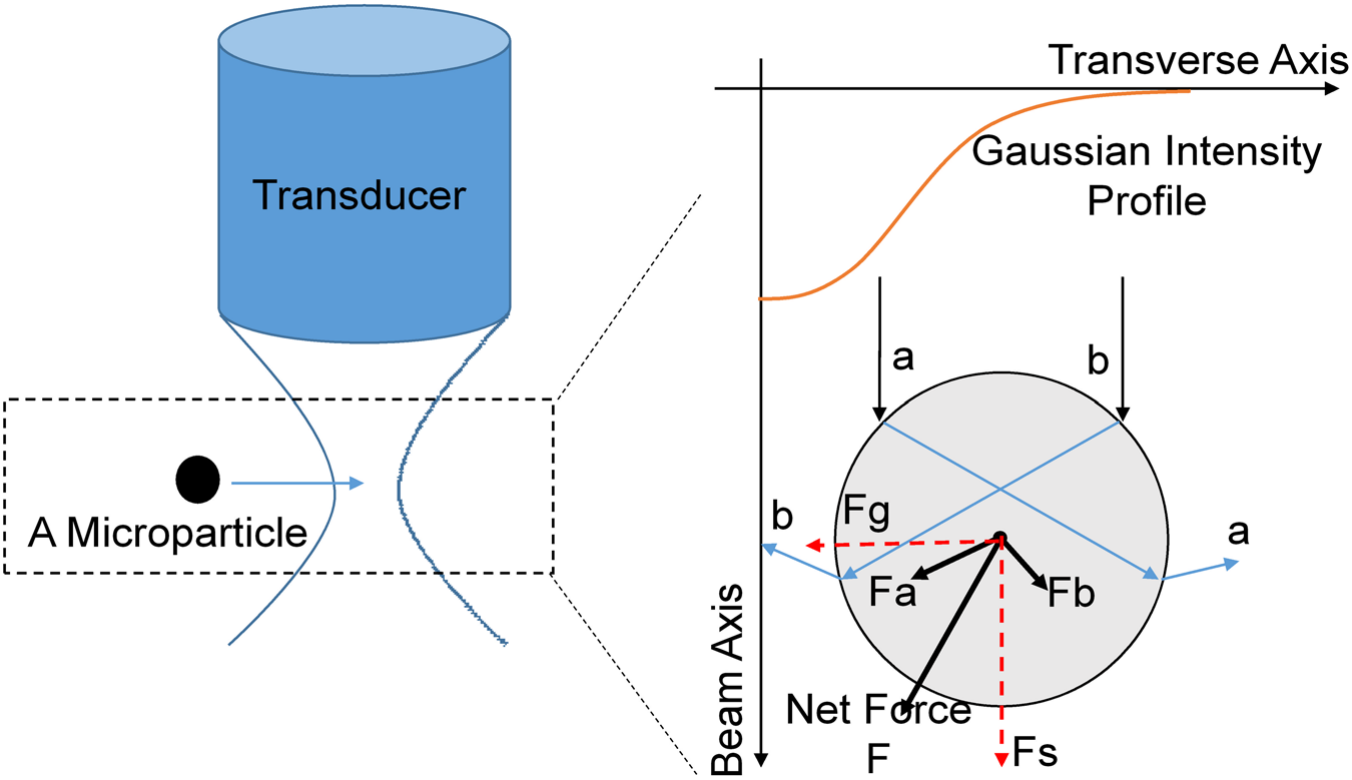
Illustration of the physics of single beam acoustic (SBA) tweezers. Two forces, scattering force (Fs) and gradient force (Fg) are generated towards the microparticle. The Fs and Fg derive from the reflection and refraction, respectively. Typically, for trapping, the gradient force needs to be larger than the scattering force.

A spherically shaped microparticle is assumed to be suspended in water for the sake of simplicity. Two incident rays (a and b) with a focused Gaussian intensity field are delivered to the microparticle, as shown in Fig. 3. Ultrasound, a mechanical wave, can be considered the motion of a series of air molecules propagating through a medium. Each of them carries a certain amount of momentum. When these molecules travel and interact with objects, the momentum transfer between molecules and objects will occur. The force striking the object from this momentum is called the radiation force. Two radiation forces, F_a_ and F_b_, are produced by a pair of rays represented by a and b on a microparticle. As a microparticle is located on an asymmetric intensity distribution, it will create an imbalanced force between F_a_ and F_b_. If F_a_ overcomes F_b_, a net force F, defined as F_g_ + F_s_, will pull the microparticle towards the beam axis. Actually, the trapping force can be viewed as a sum of two components, gradient force (F_g_) and scattering force (F_s_). The gradient force is driven by refraction, and the scattering force comes from reflection. Typically, a restoring force for SBA tweezers should be required as the gradient force is larger than the scattering force. A complete explanation for the principle of the SBA tweezers can be found in our previous publications^21,50–52^.

### Characteristics of a High-Frequency Ultrasound Transducer

A press-focused high-frequency LiNbO_3_ transducer at a center frequency of 30 MHz was fabricated. According to the simulation results using PiezoCAD software (Sonic Concepts Inc., WA, USA), the thickness of the piezo layer was lapped to approximately 100 micrometers. The Insulcast 501 epoxy (Insulcast 501; American Safety Technologies, PA, USA) and 2–3 micrometer silver particles (Silver; Aldrich Chemical Co., MO, USA) were mixed for coating on the piezo layer as the first matching layer, which was lapped to a thickness of 13 micrometers. For the backing layer, a conductive silver epoxy (E-Solder 3022; Von Roll Isola Inc., USA) was injected into a dam constructed on the other side of the piezo layer. The stack including the first matching layer, piezo layer and backing layer was placed on the center of a brass housing, and the interstice between the stack and housing was filled by epoxy (Epo-Tek 301; Epoxy Technologies, MA, USA). After sputtering with Cr/Au electrodes, a 16 micrometer parylene layer was coated on the surface of the transducer as the second layer.

**Figure 4:**
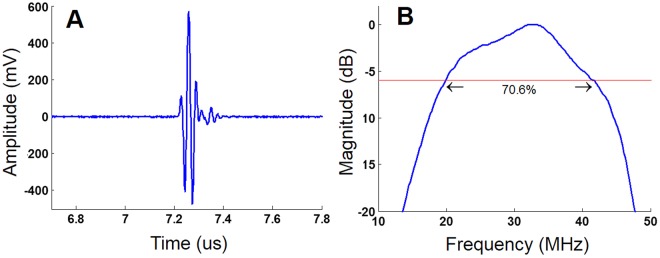
Performance of a 30 MHz high-frequency ultrasound transducer. (**A**) The pulse-echo response and (**B**) the spectrum with 70.6% bandwidth at −6 dB are represented.

The aperture diameter of 5 mm was selected by the simulation using PiezoCAD software, and a bearing ball with a diameter of 10 mm was selected to generate the focal distance of 5 mm in theory. Figure 4A shows the result of pulse-echo response, and the real focal distance was determined to be 5.4 mm by calculation. The *f*-number of 1.08 (highly focused transducer) was obtained by the definition of the focal distance divided by the aperture size. The spectrum of the pulse-echo response can be obtained by fast Fourier transform (FFT), as shown in Fig. 4B. The bandwidth at −6 dB and the Q-factor of the transducer were 70.6% and 1.466 (broad band transducer), respectively.

**Figure 5:**
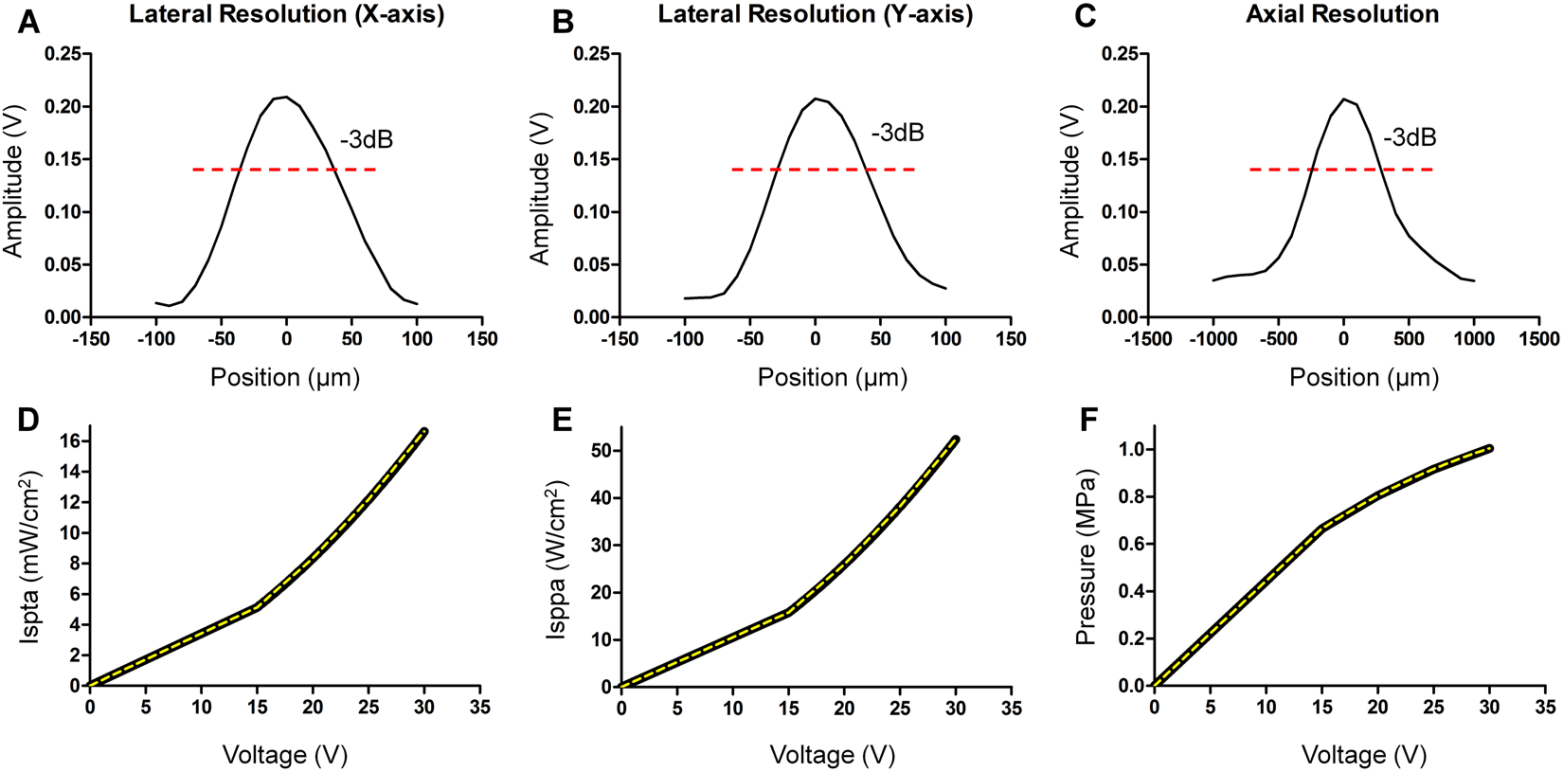
Characteristics of a 30 MHz high-frequency ultrasound transducer by hydrophone measurement. (**A**,**B**) show that the lateral resolution is approximately 60 µm in both the x-axis and in y-axis. (**C**) The axial resolution with approximately 500 µm at −3 dB is displayed. (**D**,**E**) exhibit the acoustic intensity of Ispta (mW/cm^2^) and Isppa (W/cm^2^), respectively. The acoustic pressure of the transducer is illustrated in (**F**).

A hydrophone (HGL-0085, ONDA, USA) was performed to obtain the pressure and intensity of the transducer. The 1D lateral and axial resolution profiles at −3 dB were determined to be approximately 60 and 500 micrometers, respectively, and are illustrated in Fig. 5A–C. The acoustic beam shows symmetry in both the lateral and axial direction. In addition, the spatial peak temporal average intensity (Ispta), spatial peak pulse average intensity (Isppa) and pressure were measured to quantify how much energy the transducer provides to cells. The voltages in trapping can be transferred to acoustic pressures by using the second order polynomial (quadratic) equation. A least squares curve fitting method was utilized to obtain best-fit values, and perfect fitting curves of Ispta, Isppa and acoustic pressure (yellow dash line in Fig. 5D–F) were achieved based on the following equations.

1 $${I}_{spta}=0.01239{x}^{2}+0.2087x-0.7883$$

2 $${I}_{sppa}=0.04055{x}^{2}+0.6184x-2.638$$

3 $$Acoustic\,Pressure=-\,0.0005224{x}^{2}+0.04613x+0.09023$$

where the *x* in the equation could be any voltage in trapping.

### Deformability Measurement of Suspended Cells Using The SBA Tweezer System

The position of the transducer was controlled by an XYZ positioner (SGSP 50, Sigma KOKI Co., Japan) equipped with a stage controller (SHOT-204MS, Sigma KOKI Co., Japan). To identify the focus of the transducer located on a mylar film, a pulser-receiver (5900PR, Olympus, Center Valley, PA, USA) was used to provide a signal to the transducer, and then, a tilt-based method was performed to find each maximal signal coming back from the mylar film. Before the trapping experiment, the transducer was sterilized by 70% ethanol. A function generator (SG384, Stanford Research Systems, Inc., CA, USA) was amplified in 50 dB (316.228 V) by an RF power amplifier (525LA, Electronics & Innovation, NY, USA) to drive the transducer. A duty cycle of 1% and pulse repetition frequency (PRF) of 1 KHz were set in the whole experiment. The trapping voltages from the function generator started from 0 to 70 mV with a 10 mV interval, which was associated with amplified voltages of 0, 3.16, 6.32, 9.49, 12.65, 15.81, 18.97 and 22.13 V driving to the transducer. The acoustic pressures associated with the voltage changes were 0 MPa, 0.23 MPa, 0.36 MPa, 0.48 MPa, 0.59 MPa, 0.69 MPa, 0.78 MPa and 0.855 MPa, respectively.

**Figure 6:**
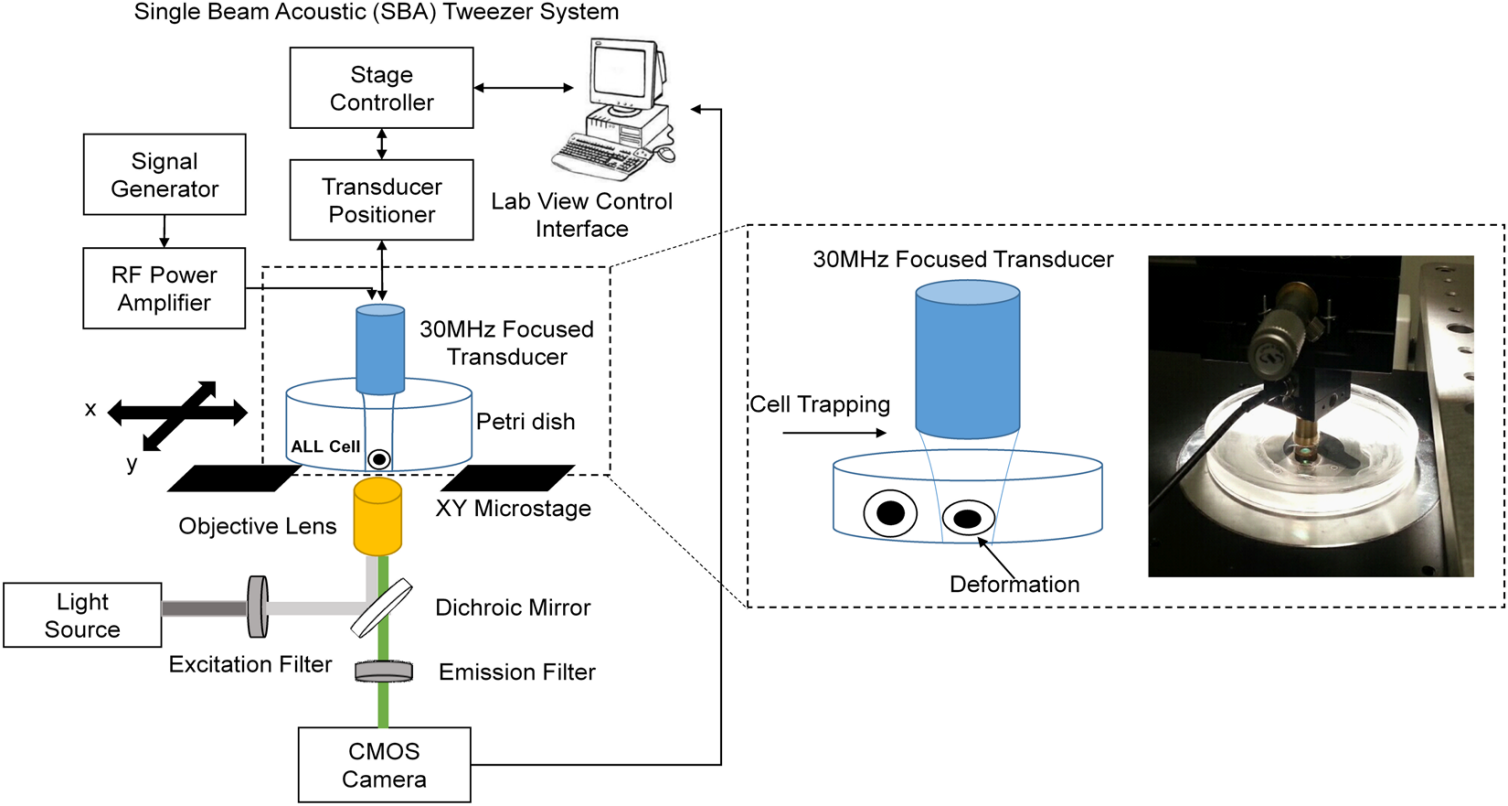
Photographic system framework of single beam acoustic (SBA) tweezers for cell deformation. A 30 MHz ultrasound transducer is mounted on an x-y-z positioner and driven by a square burst signal with various voltages transferred to a 50 dB amplifier for investigating the mechanical property of ALL cells with drug resistance.

The deformation of primary ALL and drug-resistant ALL cells was observed using an inverted microscope (IX71, Olympus, Japan) equipped with a 10X objective, and 16-bit images were recorded through a CMOS camera (C11440-10C, Hamamatsu Photonics, Japan) controlled by the software MetaMorph Version 7.7.6.0. The concentration of leukemia cells was 0.05 × 10^6^/mL. The deformability of four types of ALL cells, LAX7, LAX7R, LAX56 and ICN24, was evaluated. Each type was divided into two groups: untreated ALL cells (control group) and drug-resistant ALL cells. The SBA tweezer system for the experiment is illustrated in Fig. 6. All images were analyzed by ImageJ version 1.51j8 (National Institutes of Health, Bethesda, Maryland, USA) to delineate the boundary of the cells. The deformability is defined as follows^24^.

4 $$deformability\,of\,cells=\frac{{A}_{trapping}-{A}_{original}}{{A}_{original}}\,$$

where A_trapping_ and A_original_ represent the area of the cells after providing the acoustic radiation force to the cells with various voltages and the original size of the cells, respectively.

### Statistical Analysis

The mean and standard deviation were calculated by using Statistical Product and Service Solutions (SPSS) software version 17 (IBM, Armonk, NY, USA). A significant difference between the means of the two data groups was determined by using two-tailed unpaired *t*-tests and was defined as a *p* value < 0.05.

## Electronic supplementary material

Supplementary video 1

Supplementary video 2
